# Supplementary figures and images for: Multi-scale approaches for high-speed imaging and analysis of large neural populations
Source: PLoS Comput Biol. 2017 Aug 3;13(8):e1005685. doi: 10.1371/journal.pcbi.1005685 (PMC5557609; doi:10.1371/journal.pcbi.1005685)

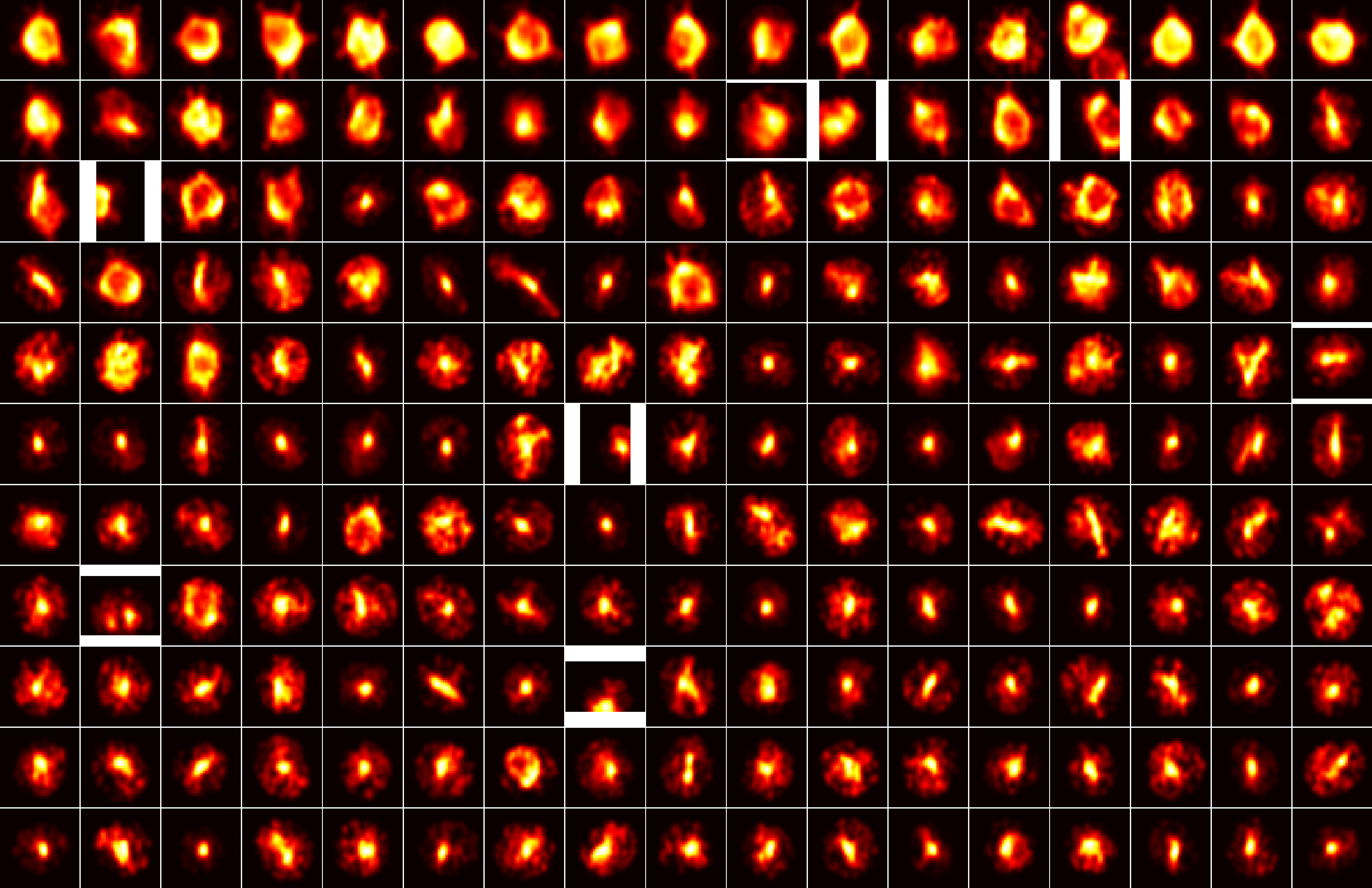

Supplement: S1 Fig — Structures that are smaller in size are more sensitive to binning. (PDF) [file pcbi.1005685.s001.pdf]

**A**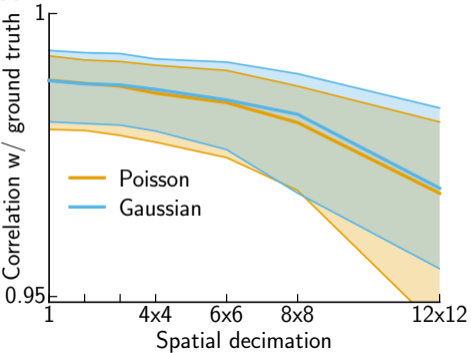**B**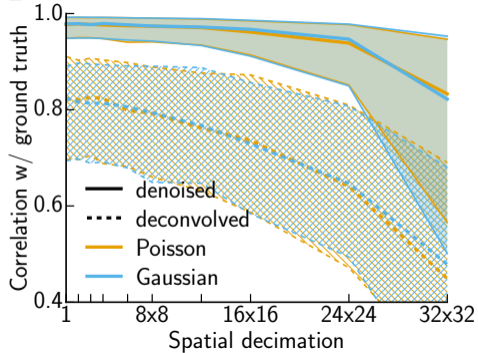**C**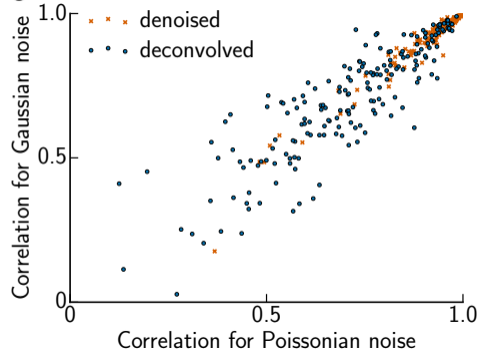

Supplement: S2 Fig — The simulated ground truth traces were obtained on decimated simulated data with Poisson (orange) or Gaussian (cyan) noise instead of reshuffling (Fig 6). (A) Correlations between denoised traces and ground truth generated from the light-sheet data. Thick lines show the median, thin lines and shaded region the IQR. (B) Correlations between denoised (solid) and deconvolved (dashed) traces and ground truth generated from the two-photon data. (C) Scatter plot showing the correlation values with ground truth for the individual traces summarized in (B). Spatial decimation was performed by averaging 16×16 pixels. (PDF) [file pcbi.1005685.s002.pdf]

**A**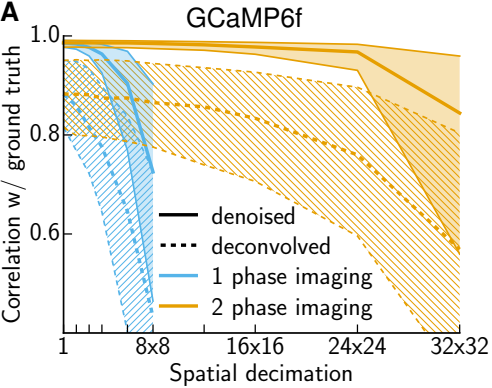**B**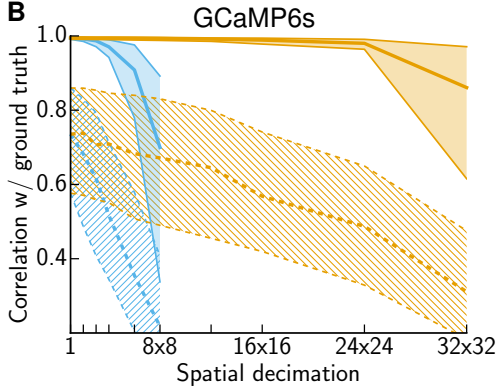

Supplement: S3 Fig — The simulated ground truth traces were obtained on decimated simulated data with Poisson noise and calcium responses that were not modeled as AR process, but instead obtained from real data using (A) GCaMP6f or (B) GCaMP6s, as discussed in the methods text. Thick lines show the median, thin lines and shaded region the IQR for denoised (solid) and deconvolved (dashed) traces. (PDF) [file pcbi.1005685.s003.pdf]
